# Emergency hysterectomy in a tertiary care hospital: indications, surgical outcomes and challenges: a 2-year retrospective descriptive cross-sectional study

**DOI:** 10.11604/pamj.2020.37.106.25393

**Published:** 2020-10-01

**Authors:** John Jude Kweku Annan, Thomas Opkoti Konney, Wilfred Sam-Awortwi, Kwasi Ampem Darkwa

**Affiliations:** 1 Department of Obstetrics and Gynaecology, Komfo Anokye Teaching Hospital, Kumasi, Ghana,; 2 School of Medicine and Dentistry, Kwame Nkrumah University of Science and Technology, Kumasi, Ghana,; 3 Department of Anaesthesiology and Intensive Care, Komfo Anokye Teaching Hospital, Kumasi, Ghana,; 4 Directorate of Obstetrics and Gynaecology, Komfo Anokye Teaching Hospital, Kumasi, Ghana

**Keywords:** Emergency hysterectomy, massive obstetric hemorrhage, placenta previa, uterine atony, uterine rupture

## Abstract

**Introduction:**

emergency hysterectomy (EH) remains a life-saving procedure in cases of life-threatening obstetric hemorrhage and other gynaecological emergencies. We aim to determine the indications, surgical outcomes and challenges of EH in our tertiary centre.

**Methods:**

an ethically approved retrospective descriptive cross-sectional study on all EHs performed at a tertiary hospital during the period of 1^st^ January 2018 to 31^st^ December 2019 was conducted. Medical records of eligible patients were retrieved, reviewed and analysed using frequencies and percentages and then summarized in tables.

**Results:**

there were 146 EHs over the two year period. The age of participants ranged from 19 to 59 years, with a mean of 34.3 years (SD = 6.06). SD: standard deviation.The main indication for EH was primary postpartum haemorrhage (PPH): 73.28% (n = 110/146). The other indications were uterine perforation with necrosis: 8.9% (n = 13/146), secondary postpartum haemorrhage: 4.8% (n = 7/146), choriocarcinoma and pelvic abscess: 2.74% (n = 4/146) each and broad ligament haematoma: 2.06% (n = 3/146). There were 3.42% (n = 5/146) which were classified as ‘others **’: two cases of ovarian cyst torsion; one case of placental site tumour; one case of incomplete septic abortion; one case of bulky multinodular fibroid uterus with severe unremitting lower abdominal pain.The most common indication for the subgroup of hysterectomy due to PPH was uterine atony 54.20% (n = 60/110), followed by ruptured uterus20.56% (n = 23/110) and then, morbidly adherent placenta 14.95% (n = 16/110). Placenta accreta constituted 62.5% (n = 10/16) of the morbidly adherent placenta.There were 91.78% (n=134/146) total abdominal hysterectomies and 8.22% (n = 12/146) subtotalhysterectomies. About eighty percent 79.45% (n = 116/146) of the surgeries required general anaesthesia, 15.07% (n = 22/146) required regional anaesthesia whilst 5.48% (n = 8/146) were started as regional anaesthesia but were converted to general anaesthesia.There were no associated intraoperative complications in 96.60% (141/146) of the cases. The most frequent intraoperative complications included bowel injury 2.04% (3/146), bladder injury 0.68% (1/146) and maternal death 0.68% (1/146).Twoof the three bowel injuries required bowel resection and anastomosis. Most of the surgeries 89.73% (n = 131/146) were performed by skilled doctors above the level of a Specialist. Major challenges faced include delayed referral of patients to the tertiary centre for prompt management and lack of quick access to blood products.

**Conclusion:**

emergency hysterectomy is performed in women who are relatively young with primary postpartum haemorrhage as the commonest indication but there are other non-obstetric indications for this emergency surgery. Though a challenging procedure, it is safe in the hands of a skilled surgical team.

## Introduction

Hysterectomy has been a common gynaecological operation for generations, and it is the most common major gynaecological operation performed worldwide [1,2], being the second most frequent surgical operation performed on women after caesarean section [3]. Hysterectomies are performed for elective and emergency indications. Emergency hysterectomies (EH) are performed due to life-threatening obstetric or gynaecologic emergencies. However, most EHs are performed for emergency obstetric indications. Emergency obstetric hysterectomy (EOH) is the extirpation of the uterus either at the time of caesarean section or following vaginal delivery, or within the puerperium as a life-saving measure in cases of intractable life-threatening obstetric hemorrhage [4-6]. Massive obstetric haemorrhage is one of the leading causes of maternal mortality and morbidity and represents the most challenging complication that an obstetrician will face [7]. Obstetric haemorrhage is a challenging condition in our sub-region withcommonest causes being uterine atony, retained placental tissue, trauma to the genital tract. In developed countries, abnormal placentation, is thought to be increasing and overtaking uterine atony as the major cause of obstetric haemorrhagebecause of the rising rate of cesarean section [8]. This trend may not be the case in developing countries as apart from the increase in the incidence of morbidly adherent placenta due to the increasing caesarean section rate, uterine atony and uterine rupture continue to be major causes of major obstetric haemorrhage. When obstetric haemorrhage occurs, certain conservative or uterine-sparing procedures can be institutedsuch asthe use of uterotonics, uterine massage, bimanual uterine compression, uterine packing, balloon tamponade, pelvic vessel ligation, B-Lynch suture, multiple square sutures, and recombinant-activated factor VII [9]. Advances in interventional radiology have also provided the option of uterine artery embolization [10,11]. In developing countries, the ready unavailability of blood products, lack of adequate infrastructure, uneasy access to interventional radiology services, lack of quick access to health care facilities and delayed referral play an important role in decision making on the best management option for obstetric haemorrhage. Therefore, EH rather becomes the intervention of first resort as the primary aim is to save the life of the woman. The unplanned nature of the surgery and the need for performing it expeditiously poses a challenge to the obstetric team. Therefore, complications though preventable, are sometimes, inevitable. Potential maternal complications include hypovolemic shock, disseminated intravascular coagulopathy, renal failure, hepatic failure, adult respiratory distress syndrome (ARDS) [11,12] and surgical complications such as injury to viscera and blood vessel and maternal death. This retrospective study was therefore aimed at examining the indications, surgical outcomes, complications and challenges of EH performed in a tertiary teaching hospital over a 2-year period (between 1^st^ January 2018 and 31^st^ December 2019). The findings will help in planning, organization of health care services, staff training and patient education to help reduce maternal morbidity and mortality from massive obstetric haemorrhage.

## Methods

**Study setting**: this retrospective descriptive cross-sectional study was conducted at the Obstetrics and Gynaecology Department of Komfo Anokye Teaching Hospital (KATH), Kumasi. KATH is a 1300 bed capacity hospital and is the second largest tertiary hospital in Ghana serving as the major referral centre for the middle and northern sectors of Ghana. It is also designated as a training and teaching facility for the Kwame Nkrumah University of Science and Technology School of Medicine and Dentistry (KNUST/KSMD), Kumasi, and offers specialised or scientific clinical care, research and teaching.

**Study population**: the scope of this study was limited to women who underwent emergency hysterectomy (EH) (obstetric and non-obstetric) over the period 1^st^ January 2018 to 31^st^ December 2019. The emergency obstetric hysterectomies (EOH) were the emergency hysterectomies performed for hemorrhage unresponsive to other therapeutic interventions at the time of delivery (cesarean section or vaginal delivery) or subsequently within the defined period of puerperium (42 days).

**Study design and data collection**: the medical records (theatre and case notes) of all women who underwent emergency hysterectomy during the study period were retrieved, reviewed and analyzed. A data capture form (proforma), designed for the purpose of this study was used to capture the data. A research assistant (a Resident in the department) was taken through training to ensure data extraction was efficient. Data extraction was conducted by the research assistant and the corresponding author. Included in the study were women who underwent emergency hysterectomy in the Obstetrics and Gynaecology Directorate of Komfo Anokye Teaching Hospital (KATH), Kumasi over the period 1^st^ January 2018 to 31^st^ December 2019. Excludedfrom the study were women who underwent emergency hysterectomy outside KATH, women who had elective hysterectomy at KATH and women who meet the inclusion criteria but have incomplete medical records. Information extracted from the medical records included socio-demographic data and surgical information: mainly age, indication for the hysterectomy, type of hysterectomy (total or subtotal). Other data were any additional procedures performed, intraoperative complications, the type of anaesthesia used and the grade of the main operator/surgeon.

**Data analysis**: the data collected was coded and entered into a pre-designed data collection proforma. No patient identifiable information was documented. The information was then entered onto a Microsoft Excel Spread sheet. The data was then cleaned and those with incomplete data excluded. The final data was then analysed using STATA version 10. The data was password protected and accessible to only the investigators. The analysis was focused on the indications and surgical outcomes of the hysterectomy.In computing the sociodemographic and surgical characteristics of the study participants, measures of central tendencies (mean, standard deviation) were used while frequencies and percentages were used to compute continuous variables and then summarized in tables.

**Ethical clearance**: ethical clearance was obtained from the Institutional Review Board for Research and Development (IRB/ R&D) of the Okomfo Anokye Teaching Hospital.

## Results

**General characteristics**: during the 2 year study period, a total of 146 emergency hysterectomies were performed. 47% (n = 69/146) in 2018 and there was a slight increase to 53% (n = 77/146) in 2019. The age of participants ranged from 19 to 59 years, with a mean of 34.3 years (SD 6.06). Women in the 30 to 39 year-old-age group constituted about 64%(n = 93/146)of cases. About 84% (n = 123/146) of cases were below 39 years old and 20.55% (n = 30/146) were below 29 years (Table 1).

**Table 1:** age distribution of women who had emergency hysterectomy

Age group(years)	Frequency(n)	Percentage (%)
<20	1	0.69
20-29	29	19.86
30-39	93	63.70
40-49	20	13.70
50-59	3	2.05
**TOTAL**	**146**	**100.00**

**Indications for emergency hysterectomy**: the various indications for the emergency hysterectomies performed were also assessed and the findings are summarized in (Table 2).The main indication for EH was primary postpartum haemorrhage (PPH): 73.28% (n = 110/146). The other indications were uterine perforation with necrosis: 8.9% (n = 13/146), secondary postpartum haemorrhage: 4.8% (n = 7/146), choriocarcinoma and pelvic abscess: 2.74% (n = 4/146) each and broad ligament haematoma: 2.06% (n = 3/146). Of the 146 emergency hysterectomies, 82.19% (n = 120/146) were emergency obstetric hysterectomies: (primary PPH, secondary PPH and broad ligament haematoma). There were 3.42% (n = 5/146) non-obstetric emergency hysterectomies which were classified as ‘others **’: 40% (n = 2/50 cases of ovarian cyst torsion; 20% (n = 1/5) case of placental site tumour; 20% (n = 1/5) case of incomplete septic abortion; 20% (n = 1/5) case of bulky multinodular fibroid uterus with severe unremitting lower abdominal pain. Subgroup analysis of those who had emergency hysterectomy for primary postpartum haemorrhage was done (Table 3). The most common indication for the subgroup of hysterectomy due to PPH was uterine atony 54.20% (n = 60/110), followed by ruptured uterus 20.56% (n = 23/110) and then, morbidly adherent placenta 14.95% (n = 16/110). There was a case (0.9%; n = 1/110) of unremitting massive obstetric haemorrhage from an extensive cervical tear that required an emergency hysterectomy. Among the morbidly adherent placenta subgroup that required emergency hysterectomy, 62.5% (n = 10/16) was due to placenta accreta, 31.25% (n = 5/16) was due to placenta percreta and 6.25% (n = 1/16) was due to placenta increta (Table 3) below depicts the results.

**Table 2:** indications for emergency hysterectomy

Indication	Frequency(n)	Percentage(%)
Primary postpartum haemorrhage	110	75.34
Uterine perforation with necrosis	13	8.90
Secondary postpartum haemorrhage	7	4.80
Choriocarcinoma	4	2.74
Pelvic abscess	4	2.74
Broad ligament haematoma	3	2.06
Others ^**^	5	3.42
**Total**	**146**	**100.00**

Others ^**^: two cases of ovarian cyst torsion; one case of placental site tumour; one case of incomplete septic abortion; one case of bulky multinodular fibroid uterus with severe unremitting lower abdominal pain

**Table 3:** indications for emergency hysterectomy due to primary postpartum haemorrhage

Indication	Frequency(n)	Percentage (%)
**Uterine atony**	60	54.55
**Uterine rupture**	23	20.91
**Morbidly adherent placenta**	16	14.55
Placenta accreta(10)		
Placenta percreta (5)		
Placenta increta(1)		
**Placenta praevia**	10	9.09
**Extensive cervical tear**	1	0.90
**Total**	**110**	**100.00**

**Surgical complications and challenges**: additional surgical procedures performed and intra-operative complications during the primary emergency hysterectomies were also assessed (Table 4). There were no associated intraoperative complications in 96.60% (n = 141/146) of the cases. The most frequent intraoperative complications included bowel injury 2.04% (n = 3/146), bladder injury 0.68% (n = 1/146) and maternal death 0.68% (n = 1/146). Two of the three bowel injuries required bowel resection and anastomosis.Additional procedures that became necessary during the index emergency hysterectomy were also assessed. No additional procedure was performed in 92.41% (n = 135/146) of cases.In 2.76% (n = 4/146) cases, unilateral salpingo-oophorectomies were performed and 2.07% (n = 3/146) cases required bilateral salpingo-oophorectomies.

**Table 4:** additional procedures performed during the emergency hysterectomy and Intra-operative complications

Additional procedure	Frequency (n)	Percentage (%)
None	135	92.41
Unilateral salpingo-oophorectomy (Right or left)	4	2.76
Bilateral salpingo-oophorectomy (BSO)	3	2.07
Bowel resection	2	1.38
Bowel repair	1	0.69
Bladder repair	1	0.69
**Total**	**146**	**100.00**
**Intraoperative complications**	**Frequency (n)**	**Percentage (%)**
None	141	96.60
Bowel injury	3	2.04
Bladder injury	1	0.68
Death	1	0.68
**Total**	**146**	**100.00**

**Surgical outcome**: of the 146 emergency hysterectomies performed, 91.78%; n = 134/146) were total abdominal hysterectomies (TAH) whilst 8.22%; n = 12) were subtotal (supracervical) hysterectomies (STAH). The method of anaesthesia administered and the grade of the main surgeon were also assessed (Table 5). About eighty percent 79.45% (n = 116/146) of the surgeries required general anaesthesia, 15.07% (n = 22/146) required regional anaesthesia whilst 5.48% (n = 8/146) were started as regional anaesthesia but were converted to general anaesthesia. Most of the surgeries 89.73% (n = 131/146) were performed by skilled doctors above the level of a Specialist. In 10.27% (n = 15) of cases, a Resident was the main surgeon.

**Table 5:** level of operator and type of operator

Type of anaesthesia	Frequency (n)	Percentage (%)
General	116	79.45
Regional	22	15.07
Regional and general	8	5.48
**Total**	**146**	**100.00**
**Operator**	**Frequency (n)**	**Percentage (%)**
Consultant	1	0.69
Senior specialist	27	18.49
Specialist	103	70.55
Resident	15	10.27
**Total**	**146**	**100.00**

## Discussion

Emergency hysterectomy (EH) is performed as a life-saving procedure to remove the uterusmainly during intractable obstetric hemorrhage or other gynaecological emergency. During the 2-year study period, a total of 146 EHs were performed in our centre; 47% (n = 69/146) in 2018 and a slight increase to 53% (n = 77/146) in 2019.Ourcentreisthe major referral tertiary centre in the middle belt of Ghana. Most of these referrals arrive in such a state that EH becomes a quick life-saving option. This accounts for the high numbers of EHs. In our series, the youngest patient was 19 years old. She presented with haemorrhagic shock from massive haemorrhage due to choriocarcinoma. The oldest patient was a 59 year old with a bulky multinodular fibroid uterus with sudden onset of severe unremitting lower abdominal pain. The mean age was34.3 years and about 84% (n = 123/146) of cases were below 39 years old. This indicates thatmore women at the prime of their reproductive age are having an abrupt end of their child-bearing potential. This has the potential of psychological morbidity on these women, especially in a society where people pride in having more children.

There are various indications for EHs. Our study showed that the commonest indication was primary postpartum haemorrhage (75.34%). Uterine perforation with necrosis constituted 8.9% of cases. Out of the 146 EHs, there were 120 peripartum hysterectomies: (primary PPH, secondary PPH and broad ligament haematoma). Of the cases of PPH, uterine atony (54.55%) was the most common indication for emergency obstetric hysterectomy (EOH). This was followed by uterine rupture (20.91%) and morbidly adherent placenta (14.55%). This reflects the situation in most developing countries where atony accounts for the majority of cases of EOH. Studies by Varras *et al* in 2010 [13], Rabiu *et al*. 2010 in Nigeria [14] and from other tertiary care centers in India, [15] the United Kingdom [16] and Turkey [17] also support this. It is therefore imperative to address measures to reduce risk factors for uterine atony such asgrand multiparity, injudicious oxytocin use for uterine stimulation, multiple pregnancy and prolonged labour. Uterine rupture was a significant contributor to EOH: constituting20.91% of indications in our series. Risk factors for uterine rupture include a scarred uterus, grandmultiparity and prolonged unsupervised labour. These factors are prevalent in our setting. In the United Kingdom, according to a UKOSS study, uterine rupture leads to EOH in 8% of cases [16]. In Turkey, uterine rupture constituted close to 17% [17]. These rates are lower than that of our study. Morbidly adherent placenta was the third (14.55%) leading cause of EOH in our series. Among this subgroup, 62.5% (n = 10/16) was due to placenta accreta, 31.25% (n = 5/16) was due to placenta percreta and 6.25% (n = 1/16) was due to placenta increta. Other studies foundmorbidly adherent placenta and placenta previaas the primary etiological causes for EOH [18,19]. Other studies also found placenta accreta as the primary indication in these women and accounts for 38%-50% of all peripartum hysterectomies [5, 20-22]. Therefore, the obstetrician must prepare for the possibility of EOH in these patients. Antenatal detection of morbidly adherent placenta by using Doppler ultrasound and confirmation with magnetic resonance imaging (MRI) and the introduction of interventional radiology services are recommended in these women. However, in low-resourced countries, these facilities are not readily available. Even when they are available, and extensive experience needed preclude their use. A multidisciplinary team approach to the management of these women is recommended [23].

In the initial management of massive obstetric haemorrhage, uterine conserving measures such as administration of uterotonic drugs, uterine or hypogastric artery embolisation, insertion of hemostatic sutures, and uterine or internal iliac artery ligation are employed. These measures are of particular importance in patients who are young, have low parity and who are haemodynamically stable. In situations where conservative treatment is likely to fail or has failed, there should be no further delay in performing EH. Once EH is decided on, the obstetrician is faced with another challenge: the choicebetween subtotal and total abdominal hysterectomy. Our series foundthat total abdominal hysterectomy (TAH) was the most commonly performed surgical procedure: (91.78%) compared to (8.22%) of subtotal hysterectomies (STAH).This finding is at variance with the findings of other studies that found STAH as the commonest [5,24-26]. Proponents of STAH report a lesser blood loss, a reduced need for blood transfusion, reduced operating time in the face of hemodynamic compromise/instabilityand reduced intra and postoperative complications such as fewer instances of damage to the urinary tract [27,28]. However, TAH is the recommended surgical method of EPH. It has the advantage of removing the development of malignancy in the cervical stump, the need for regular cytology and other associated problems such as bleeding or discharge associated with the residual cervical stump. Additionally, TAH is effective in the management ofmorbidly adherent placenta located in the lower uterinesegment as removal of the cervix leads to better hemostasis.However, STAH may be a better choice in certain conditions where surgery needs to be completed in a shorter time.

During EOH,the procedure needs to be performed quickly to arrest the bleeding and save the life of the woman. This may lead to complications such as injury to the urinary tract (bladder and ureters), bowel and blood vessels. In our series, showed low complication rates. There were 2.04% (n = 3/146) cases of bowel injury. Two of these required bowel resection and end to end anastomosis. There was 0.68% (n = 1/146) case of bladder injury which was repaired and0.68% (n = 1/146) of intraoperative mortality. These low complication rates can be attributable to the level of expertise and experience of the primary surgeon. In about 90% of the cases,the primary surgeon was a Specialist or Senior Specialist/Consultant assisted by residents in training. Even the 10% that were performed by Residents in Obstetrics and Gynaecology, those cases were under the direct supervision of a more senior obstetrician.This has an advantage of improving training in the acquisition of the requisite skills of the trainees whilst not compromising on the quality of care offered to these women.There is evidence of significant reduction in operating time, number of units of blood transfusion and hospital stay whenEPH is performed by an experienced surgeon [29]. General anaesthesia was the most popular method of anaesthesia (80%). Regional anesthesia was used in 15% of cases. In 5% of the cases, regional anaesthesia was converted to general anaesthesia. Due to the excessive bleeding, the potential for other additional procedures and in order to reduce patient anxiety, general anaesthesia wasour preferred option albeit its own potential complications in a patient who has not been assessed fully and prepared adequately for surgery.

## Conclusion

Emergency hysterectomy, even though it curtails the child bearing potential of a woman, is indicated as a life-saving procedure in women who are relatively young with unremitting life-threatening primary postpartum haemorrhage and other gynaecological indications.Though a challenging procedure, the involvement of an experienced surgical team in its management is paramount in reducing morbidity and mortality.There are limitations to this study. It is a retrospective study design and the data collected were from a single institution. As such, the above conclusions cannot be generalized.

### What is known about this topic

- Emergency hysterectomy (EH) remains a life-saving procedure in cases of severe intractable life-threatening obstetric hemorrhage and other gynaecological emergencies;
- Reduced caesarean section and multiple pregnancy rates, availability of adequate infrastructure, blood products and interventional radiology services help in the optimum management of life-threatening obstetric haemorrhage.

### What this study adds

- In low-resourced countries, in young women, emergency hysterectomy is indicated as an effective first line life-saving intervention in severe intractable haemorrhage;
- Complication rates of emergency hysterectomy are very low in the hands of an experienced and skilled surgical team.
